# Effects of a person-centred, nurse-led follow-up programme on adherence to prescribed medication among patients surgically treated for intermittent claudication: randomized clinical trial

**DOI:** 10.1093/bjs/znac241

**Published:** 2022-07-15

**Authors:** Sara T Haile, Eva Joelsson-Alm, Unn Britt Johansson, Helena Lööf, Ulrika Palmer-Kazen, Peter Gillgren, Anneli Linné

**Affiliations:** Department of Clinical Science and Education, Södersjukhuset, Karolinska Institutet, Stockholm, Sweden; Department of Surgery, Section of Vascular Surgery, Södersjukhuset, Stockholm, Sweden; Department of Clinical Science and Education, Södersjukhuset, Karolinska Institutet, Stockholm, Sweden; Department of Anaesthesiology and Intensive Care, Södersjukhuset, Stockholm, Sweden; Department of Clinical Science and Education, Södersjukhuset, Karolinska Institutet, Stockholm, Sweden; Department of Health Promoting Science Sophiahemmet University, Stockholm, Sweden; Department of Health Promoting Science Sophiahemmet University, Stockholm, Sweden; Division of Caring Sciences, School of Healthcare and Social Welfare, Mälardalen University, Västerås, Sweden; Department of Molecular Medicine and Surgery, Karolinska Institutet, Stockholm, Sweden; Department of Vascular surgery, Karolinska University Hospital, Stockholm, Sweden; Department of Clinical Science and Education, Södersjukhuset, Karolinska Institutet, Stockholm, Sweden; Department of Surgery, Section of Vascular Surgery, Södersjukhuset, Stockholm, Sweden; Department of Clinical Science and Education, Södersjukhuset, Karolinska Institutet, Stockholm, Sweden; Department of Surgery, Section of Vascular Surgery, Södersjukhuset, Stockholm, Sweden

## Abstract

**Background:**

Management of intermittent claudication should include secondary prevention to reduce the risk of cardiocerebrovascular disease. Patient adherence to secondary prevention is a challenge. The aim of this study was to investigate whether a person-centred, nurse-led follow-up programme could improve adherence to medication compared with standard care.

**Methods:**

A non-blinded RCT was conducted at two vascular surgery centres in Sweden. Patients with intermittent claudication and scheduled for revascularization were randomized to the intervention or control (standard care) follow-up programme. The primary outcome, adherence to prescribed secondary preventive medication, was based on registry data on dispensed medication and self-reported intake of medication. Secondary outcomes were risk factors for cardiocerebrovascular disease according to the Framingham risk score.

**Results:**

Some 214 patients were randomized and analysed on an intention-to-treat basis. The mean proportion of days covered (PDC) at 1 year for lipid-modifying agents was 79 per cent in the intervention and 82 per cent in the control group, whereas it was 92 *versus* 91 per cent for antiplatelet and/or anticoagulant agents. The groups did not differ in mean PDC (lipid-modifying *P* = 0.464; antiplatelets and/or anticoagulants *P* = 0.700) or in change in adherence over time. Self-reported adherence to prescribed medication was higher than registry-based adherence regardless of allocation or medication group (minimum *P* < 0.001, maximum *P* = 0.034). There was no difference in median Framingham risk score at 1 year between the groups.

**Conclusion:**

Compared with the standard follow-up programme, a person-centred, nurse-led follow-up programme did not improve adherence to secondary preventive medication. Adherence was overestimated when self-reported compared with registry-reported.

## Teaser

Management of intermittent claudication should include secondary prevention to reduce the risk of cardiocerebrovascular disease. This RCT aimed to investigate whether a person-centred, nurse-led follow-up programme, compared with standard care, after surgery could increase adherence to lipid-modifying and antiplatelet/anticoagulant agents. The results showed no difference between the intervention and standard follow-up programme on terms of patients’ adherence to secondary preventive medication.

## Background

Intermittent claudication (IC) is associated with an increased risk of cardiocerebrovascular disease, and can be an early predictor of cardiovascular-related mortality^1–5^. To reduce this risk, the management of IC should include secondary prevention. Secondary prevention comprises pharmacological treatment, lifestyle changes, most significantly smoking cessation, and increased physical activity^1,4,6^. Patients with claudication should be treated with lipid-modifying agents, antiplatelet agents, and antihypertensives for those with hypertension^1,2,6,7^. Patients’ adherence to prescribed medication and preventive measures is essential in achieving risk reduction; non-adherence is associated with increased long-term cardiocerebrovascular events and mortality^8–10^. Non-adherence to medication is widespread and a major concern for healthcare professionals^11,12^. Although medication adherence is important for individuals with long-term conditions, up to 50 per cent of all patients have poor adherence to long-term therapy^12^. Adherence to a medication regimen that prevents cardiovascular diseases has been reported to be as low as 48 per cent^10^, 30 per cent^13^, and 57 per cent^14^.

Although the concept of adherence is mostly associated with adherence to a medication regimen, it can also reflect other therapeutic behaviours, such as seeking medical attention and implementing behavioural modifications, including smoking cessation or increased physical activity^11^.

A review^15^ of 27 trials including a total of 899 068 patients concluded that different interventions with either a team-based healthcare system, intensified patient care such as electronic reminders, patient education by healthcare professionals, or pharmacist-led intervention could improve adherence to lipid-lowering medicines. Interventions such as multifaceted support after treatment for acute coronary syndrome and nurse-delivered self-care training after hospitalization for heart failure have been found to improve adherence to medication compared with standard care^13,16^. In observational trials^17,18^ evaluating nurse-led risk management among patients with IC, improvements in total cholesterol, heart risk score, and health-related quality of life were observed. Similar results were reported in another trial^19^ in which web-based or live counselling on lifestyle and medication after acute coronary syndrome were evaluated. Adherence among patients with IC 3 months after revascularization has been reported to be 81 and 77 per cent to anticoagulant and statin medication regimens respectively^20^. Additionally, adherence to a medication regimen has been shown to decrease over time^21^. Among patients who received an intervention for acute coronary syndrome, non-adherence was reported to increase from 20 per cent immediately after an intervention to 54 per cent at 6 months and 53 per cent after 1 year^21^. Only complex measures including composite actions were found to improve adherence to long-term treatments, unlike in short-term treatments, where single measures were sufficient^22^.

There is a growing call from national authorities for healthcare providers to offer more person-centred care, that is involvement of the patient as a partner in their care and individualized measures^23,24^. It has been suggested that patient-centred care could lead to better adherence and improved outcomes^24^. This study aimed to examine the effects of a person-centred, nurse-led follow-up programme on patients’ adherence to prescribed lipid-modifying and antiplatelet/anticoagulant medication, compared with those of typical care, and the effects of such a programme on risk factors associated with cardiocerebrovascular events.

## Methods

### Study design and participants

This multicentre RCT compared a person-centred, nurse-led follow-up intervention programme with standard follow-up care (FASTIC study). In Sweden, claudication is primarily treated conservatively with exercise, lifestyle changes (smoking cessation), and best medical treatment, usually at a primary care facility. For patients who have persisting severe claudication in spite of these efforts, revascularization can be considered after referral to a vascular centre. Revascularization is planned depending on the location of vascular lesions, and can be open or endovascular. Generally, smoking cessation for at least 3 months is required. All patients should be prescribed both an antiplatelet agent and a lipid-modifying agent, unless contraindicated. Antihypertensive agents are prescribed in accordance with European guidelines. After revascularization, patients who undergo endovascular treatment and receive a stent, placed in the superficial femoral artery or more distally, are in addition prescribed dual antiplatelet treatment for 1–6 months.

Patients were recruited at the two large hospitals conducting vascular surgery in Stockholm, Sweden. All patients diagnosed with IC (ICD-10 codes I70.2 or I739B) scheduled for vascular surgery (open and/or endovascular) from June 2016 to October 2018 (centre 1) or from September 2017 to November 2018 (centre 2) were screened for eligibility. Inclusion criteria were age at least 18 years, absence of signs of critical limb ischaemia (rest pain, ulceration, or gangrene), and ability to speak and understand the Swedish language. Exclusion criteria were: dementia, planned discharge to a nursing home, not being accountable for administrating own medications, or a survival expectancy of less than 1 year. All eligible patients were invited to participate in the study and written informed consent was obtained. There was no difference between the two centres in expertise or standard care of patients with IC. The number of patients treated differed between the two centres owing to the later study start at centre 2.

### Randomization

**Fig. 1.**
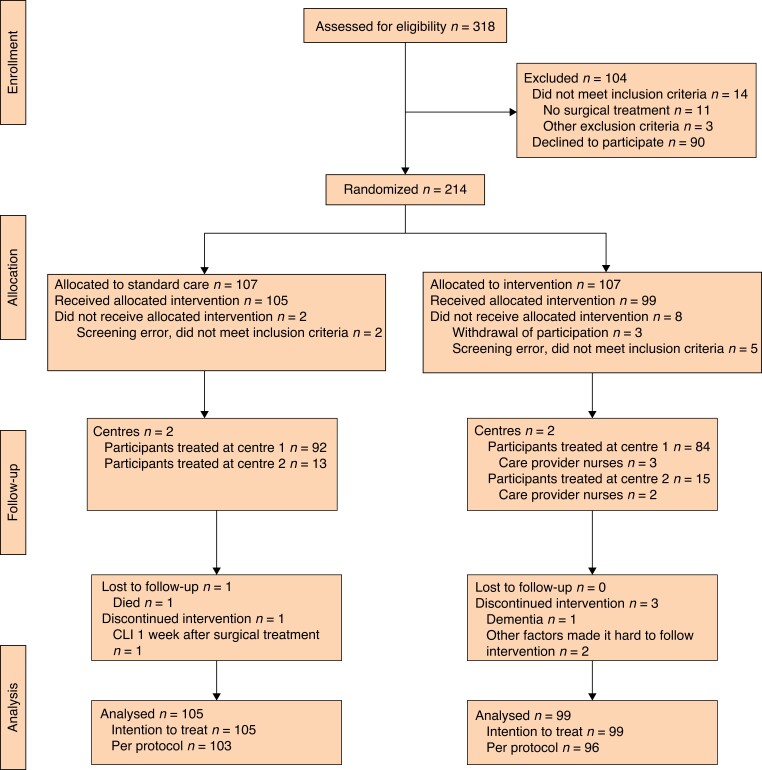
CONSORT diagram for the study CLI, critical limb ischaemia.

Participants were randomized to either a person-centred, nurse-led follow-up programme (intervention group), or a standard care follow-up programme (control group). Randomization was achieved by sequence generation using secure computer-generated random numbers, by a study nurse who was unaware of the block randomization factors and the sequence generation process. In some instances, where a planned endovascular procedure may not have resulted in revascularization and no further surgical treatment was planned, the patient was withdrawn from the study (*Fig. 1*). No blinding was applied in this study. Details of the enrolment procedure can be found in the published study protocol^25^.

### Intervention and standard care programmes

The intervention consisted of a person-centred, nurse-led programme, including three visits and two telephone calls undertaken by a specially trained vascular nurse during the first year after revascularization. The person-centred care model used was that of the Gothenburg centre for person-centred care, developed at the University of Gothenburg, comprising the establishment of a partnership between the professional healthcare worker and the patient; patient narratives; and a documented self-care plan with goals, self-care activities, and a plan for future follow-up and revision^23^. The standard care programme included two visits during the first year after revascularization, one to a vascular surgeon 4–8 weeks after surgery and another to either a vascular surgeon or a vascular nurse at 1 year. The same counselling was given regardless of the type of vascular surgery performed. The study protocol, with detailed information on the study design, procedure, and the content of the person-centred, nurse-led programme and standard care, has been published^25^.

### Sample size

The hypothesis was that the intervention programme would increase adherence to the prescribed medication regimen from 50 to 70 per cent, based on the results of previous studies in cardiology^13,26^. A sample size calculation indicated that a total of 186 participants was needed to detect a statistically significant increase in adherence to prescribed medication from 50 to 70 per cent (power 0.80, significance level 0.05, 2-sided). The statistical power analysis was done using SPSS^®^ version 25, Sample Power 3 (IBM, Armonk, NY, USA).

### Data collection and outcome measures

#### Primary outcome: adherence to prescribed medication

Adherence to the prescribed medication regimen was defined as the proportion of days covered (PDC), and calculated by dividing the number of available dispensed doses (registry data) by the number of days the patient was prescribed the medication (data from medical records).

**Fig. 2.**
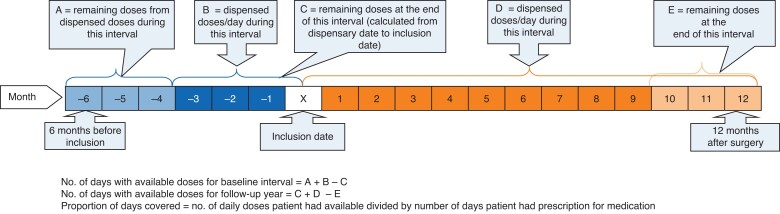
Calculation of doses available at home during the study

The number of available dispensed doses was collected from a registry at Region Stockholm, Centre for Health Data, a local registry that reports to the national Swedish prescribed drug registry^27,28^. Data regarding each participant’s retrieved doses of all lipid-modifying, antiplatelet, and anticoagulant agents were collected for 18 months (6 months before the date of inclusion to 12 months after revascularization) from the registry for each participant. The WHO Collaborating Centre for Drug Statistics Methodology classification of Anatomical Therapeutic Chemical codes was used to identify antiplatelet, anticoagulant, and lipid-modifying agents in the registry (*Table 1*). The number of available doses at home was calculated for each medicine in the baseline interval (3 months before inclusion) and 1 year follow-up (12 months following vascular surgery). In addition, patients were asked about their adherence. No other measure of control was used. Doses available in the baseline interval were calculated by the addition of all dispensed doses between the date of inclusion and the 3-month interval preceding that date. For the follow-up phase, doses available were calculated by adding every dispensary between the date of surgery and 12 months after surgical treatment. Doses available at home from previous dispensaries were added for each interval. The calculation of doses available is further described in *Fig. 2*. The number of days with prescribed medication was adjusted for hospital admissions occurring during the study interval as prescribed medicines were then supplied by the hospital.

**Table 1:** Medications included in study with Anatomical Therapeutic Chemical codes according to the WHO Collaborating Centre for Drug Statistics Methodology classification

Medication group	ATC code
**Antiplatelet and/or anticoagulants**	
** **Vitamin K antagonists	B01AA
** **Heparin group	B01AB
** **Platelet aggregation inhibitors excluding heparin	B01AC
** **Direct thrombin inhibitors	B01AE
** **Direct factor Xa inhibitors	B01AF
**Lipid-modifying agents**	
** **HMG CoA reductase inhibitors	C10AA
** **Fibrates	C10AB
** **Other lipid-modifying agents	C10AX

ATC, Anatomical Therapeutic Chemical; HMG CoA, 3-hydroxy-3-methylglutaryl co-enzyme A.

After calculating the number of days that the patient was prescribed each medicine and the number of doses they had available, a total sum was calculated for each group of medications (antiplatelets, anticoagulants, and lipid-modifying agents). Adherence as PDC was analysed both as continuous mean proportion, and as dichotomized data whereby patients with a PDC of at least 80 per cent were classified as being adherent, and the rest as non-adherent.

Self-reported adherence was assessed at baseline and at 1 year through study-specific multiple-choice questions to evaluate how often the patient had taken the specific medication during the past month. Possible answers were: 7 days a week, 5–6 days a week, 3–4 days a week, 1–2 days a week, ‘I have no prescription of the medication,’ or ‘I don’t know if I am prescribed the medication’.

To compare self-reported with registry-reported adherence, the self-reported adherence to medication (range of days per week) was converted to the PDC per year.

#### Secondary outcomes

Baseline data on co-morbidities and pharmacological treatment were based on patient recollection and medical records. The Framingham risk score (FRS) was used to assess the predicted 10-year risk of cardiocerebrovascular events. The total point and risk estimation were based on an algorithm that included sex-specific general risk factors (age, systolic BP, treated/untreated hypertension, smoking status, diabetes, level of total cholesterol, and high-density lipoprotein)^29–31^.

The total Framingham score was calculated at baseline and 1 year. Age was adjusted for by adding 1 to the age recorded at baseline when calculating the total score at 1 year. For the variable difference over time in FRS, the total score at baseline was subtracted from the total score at 1 year.

Serum blood samples were obtained to assess levels of total cholesterol, high- and low-density lipoproteins, triglycerides, and haemoglobin (Hb) A1c.

Systolic and diastolic BP at rest, weight (for calculation of BMI), and waist circumference were measured at study enrolment and 1 year after revascularization. Ankle brachial pressure index (ABPI) was measured and calculated at study enrolment, 1 day after revascularization, and at 4–8 weeks, and 1 year after revascularization. The data were analysed based on treatment goals recommended by guidelines as follows: systolic BP less than 140 mmHg, diastolic blood pressure less than 90 mmHg, low-density lipoprotein no more than 1.8 mmol/l, total cholesterol no more than 5 mmol/l, and HbA1c no more than 52 mmol/mol for patients with diabetes and no more than 46 mmol/mol for patients without diabetes^1,16^.

Smoking status was assessed according to an assessment instrument developed by the Swedish National Board of Health and Welfare^32^ at study enrolment, after 4–8 weeks, and at 1 year. Patients were asked to state their perceived pain-free walking distance in metres. Change in pain-free walking distance was analysed on the basis of the reported distance at the first revisit (4–8 weeks) and 1 year in an attempt to avoid measuring primarily improvement expected owing to the vascular surgery.

Data regarding type of vascular surgery performed, and complications were collected from the local quality registry (Swedvasc).

### Statistical analysis

Normally distributed continuous data are presented as mean(s.d.), and skewed data as median (i.q.r.). The Shapiro–Wilk test was used to check the normality of data distribution. Categorical data are presented as number and percentage. To compare differences between groups, the Pearson χ^2^ or Fisher’s exact test was used for proportions, the independent-samples Mann–Whitney *U* test for skewed data, and the independent-samples *t* test for normally distributed data. The related-samples McNemar test was used to compare differences between self-reported and registry-reported adherence to medication. Repeated-measures ANOVA was used to test the change over time between two measurement points (baseline and 1 year). The proportion of patients within the treatment goal in each group was analysed at 1 year. Differences between the two groups over time were evaluated based on improvement toward, or deterioration from, a treatment goal. If the result at 1 year for the whole group was improved, the analysis was based on those patients who were not within the treatment goal at baseline (with a chance of improvement). If the result at 1 year for the whole group had deteriorated over time, those within the treatment goal at baseline (at risk of deteriorating) were included in the analysis. The intention-to-treat principle was applied during the analysis. Two-tailed *P* < 0.050 was considered statistically significant. All statistical analyses were carried out using SPSS^®^ version 28.

## Ethics

The study was approved by the Regional Ethical Review Board in Stockholm (registration number 2015/2346-31/2) and was registered at ClinicalTrials.gov (NCT03283358). Written and oral informed consent was obtained from all participants. The trial was conducted in compliance with the Helsinki Declaration and reported in accordance with the CONSORT statement^33–35^.

## Results

A total of 318 patients were assessed for eligibility (*Fig. 1*). Ninety patients declined to participate; the most common reason stated was the long travel distance from the hospital. Fourteen patients were excluded because no intervention was performed (11) and owing to other exclusion criteria (3). A total of 214 patients consented to participate and were randomized to either the intervention group (107) or the control group (107). After randomization, 2 patients in the control group were excluded because of screening error (not meeting inclusion criteria), and 8 patients in the intervention group owing to withdrawal of consent (3) or screening error (not meeting inclusion criteria (5). A total of 204 patients remained, of whom 176 were treated at centre 1 and 28 at centre 2. A total of five patients did not fulfil the study protocol (*Fig. 1*). The groups were comparable at baseline (Table 2, *Table S1*). Based on data from medical records, 99.0 per cent of patients in both groups (98 of 99 and 104 of 105) were prescribed antiplatelet medications and/or anticoagulants, whereas 91 of 99 (92 per cent) in the intervention group and 101 of 105 (96.2 per cent) in the control group were prescribed lipid-modifying agents (*Table 2*).

**Table 2:** Baseline characteristics of study population

	Person-centred, nurse-led care (*n* = 99)	Standard care (*n* = 105)
**Demographics**		
Age (years), median (i.q.r.)	71 (66 to 76)	72 (69 to 77)
Women	40 (40)	54 (51.4)
Smoking status*		
Never smoker	8 (8)	8 (7.6)
Current smoker	5 (5)	4 (3.8)
Previous smoker	86 (87)	93 (88.6)
Stopped ≥ 6 months before inclusion	69 (70)	77 (73.3)
Stopped < 6 months before inclusion	17 (17)	16 (15.2)
**Co-morbidity†**		
Ischaemic heart disease	34 (34)	31 (29.5)
Heart failure	4 (4)	10 (9.5)
Hypertension	83 (84)	91 (86.7)
Cerebrovascular disease	15 (15)	16 (15.2)
COPD	17 (17)	19 (18.1)
Chronic renal failure	1 (1)	3 (2.9)
Diabetes mellitus	32 (32)	34 (32.4)
Previous peripheral vascular surgery	33 (33)	37 (35.2)
**Medical treatment†**		
Antiplatelets and/or anticoagulants	98 (99)	104 (99.0)
Lipid-modifying agent	91 (92)	101 (96.2)
Antihypertensives	85 (86)	92 (87.6)
**Baseline measurements**		
Pain-free walking distance (m), median (i.q.r.) (missing *n* = 2|2)*	100 (50 to 150)	100 (50 to 200)
0–200	86 (89)	86 (83.5)
200–500	11 (11)	17 (16.5)
≥ 500	0 (0)	0 (0)
ABPI, median (i.q.r.)	0.59 (0.47–0.75)	0.57 (0.47 to 0.72)
BMI (kg/m^2^), mean(s.d.) (missing *n* = 2|0)	27.1(4.4)	27.2(4.3)
LDL, patients within treatment goal (≤ 1.8 mmol/l) (missing *n* = 3|2)	46 (48)	55 (53.4)
Total cholesterol, patients within treatment goal (≤ 5 mmol/l)	81 (82)	89 (84.8)
Systolic BP, patients within treatment goal (< 140 mmHg)	38 (38)	32 (30.5)
Diastolic BP, patients within treatment goal (< 90 mmHg) (missing *n* = 1|2)	89 (91)	96 (93.2)
HbA1c, patients within treatment goal (≤ 52 mmol/mol with diabetes, ≤ 46 mmol/mol without diabetes) (missing *n* = 16|18)	56 (68)	68 (78)

Values are *n* (%) unless otherwise indicated. Numbers of patients with missing data are shown for intervention group|control group. *Self-reported; †data from medical records. ABPI, ankle brachial pressure index; COPD, chronic obstructive pulmonary disease; LDL, low-density lipoprotein; HbA1c, haemoglobin A1c.

### Adherence to medication according to registry

There were no differences in mean adherence (PDC) at baseline between the intervention and control groups regarding lipid-modifying agents (84 *versus* 85 per cent; *P* = 0.729) or antiplatelets and/or anticoagulants (90 *versus* 89 per cent; *P* = 0.593).

**Fig. 3.**
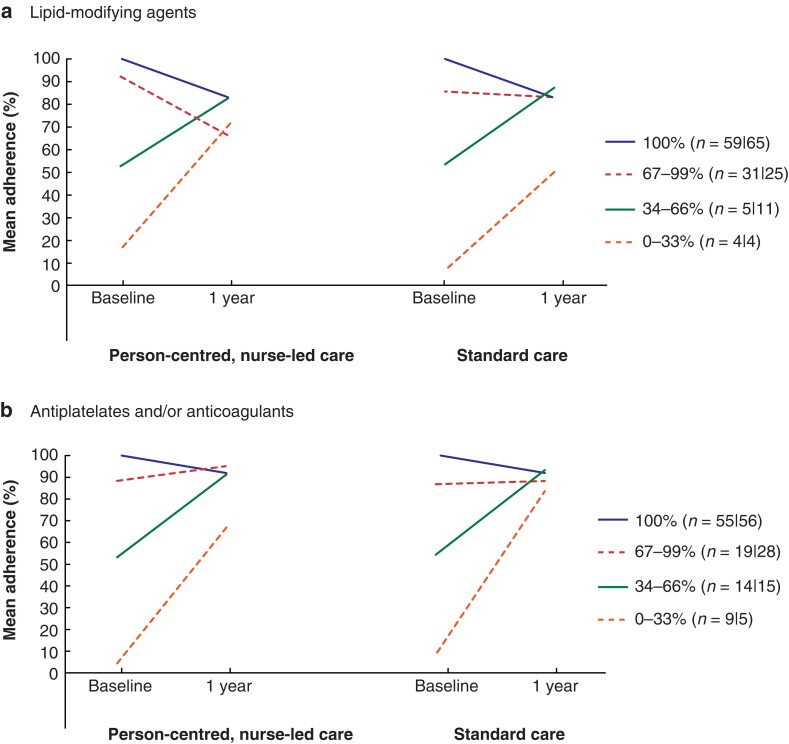
Adherence over time for subgroups according to mean adherence to lipid-modifying agents or antiplatelet and/or anticoagulant agents at baseline **a** Lipid-modifying agents and **b** antiplatelet and/or anticoagulant agents. Mean differences over time for intervention *versus* control group: **a**  *P* = 0.870, *P* = 0.206, *P* = 0.742, and *P* = 0.138 for subgroups with 100, 67–99, 34–66, and 0–33 per cent adherence at baseline respectively; **b**  *P* = 0.957, *P* = 0.194, *P* = 0.787, and *P* = 0.178 for subgroups with 100, 67–99, 34–66, and 0–33 per cent adherence at baseline respectively (Repeated-measures ANOVA).

For both groups, mean adherence to lipid-modifying agents tended to decrease over time (−5 *versus* −3 percentage points for intervention and control groups respectively; *P* = 0.467), whereas it tended to increase over time for antiplatelets and/or anticoagulants (+2 *versus* +2 percentage points; *P* = 0.551) (*Table 3*). At baseline, 100 per cent adherence was achieved by 111 of 201 patients for lipid-modifying agents, and by 124 of 204 for antiplatelets and/or anticoagulants. Changes in adherence over time for subgroups based on mean adherence at baseline are shown in *Fig. 3.*

**Table 3:** Primary outcome: adherence to prescribed medication regimen according to data from prescribed drug registry from Centre for Health Data, Stockholm, Sweden

	Baseline		1 year		*P*‡
	Person-centred, nurse-led care (*n* = 99)	Standard care (*n* = 105)	Person-centred, nurse-led care (*n* = 99)	Standard care (*n* = 105)	
**Lipid-modifying agents**	*n* = 97	*n* = 104	*n* = 97	*n* = 104	
Adherence (%), mean(s.d.)	84(28)	85(25)	79(27)	82(27)	0.464
Difference in adherence (1 year – baseline) (%), mean(s.d.)			−5(37)	−3(33)	0.467
Patients with PDC ≥ 80%	73 (75)	76 (73.1)	61 (63)	73 (70.2)	0.297
* * Deteriorated from PDC ≥ 80% (*n* =73|76)*			27 (37)	19 (25)	0.156
**Antiplatelets and/or anticoagulants**	*n* = 99	*n* = 105	*n* = 99	*n* = 105	
Adherence (%), mean(s.d.)	90(21)	89(22)	92(14)	91(19)	0.700
Difference in adherence (1 year – baseline) (%), mean(s.d.)			2(21)	2(27)	0.551
Patients with PDC ≥ 80%	85 (86)	83 (79.1)	83 (84)	90 (85.7)	0.846
* * Reached PDC ≥ 80% (*n* = 14|22)†			8 (57)	16 (73)	0.471

Values are *n* (%) unless otherwise indicated. Numbers of patients for whom data are available shown for intervention group|control group. *Of those who had proportion of days covered (PDC, number of available dispensed doses (registry data) by the number of days the patient was prescribed the medication (data from medical records)) over 80 per cent at baseline. †Of those who had PDC less than 80 per cent at baseline. 1 year, surgical treatment + 12 months. ‡Repeated-measures ANOVA test.

Mean adherence (PDC) at 1 year for lipid-modifying agents was 79 per cent in the intervention group and 82 per cent in the control group, whereas it was 92 *versus* 91 per cent for antiplatelet medications and/or anticoagulants. There were no differences between the groups either in mean PDC (lipid-modifying *P* = 0.464; antiplatelets and/or anticoagulants *P* = 0.700) or in the change in adherence over time (*Table 3*).

With a cut-off level of 80 per cent PDC considered adherent, there were no significant differences between the two groups regarding lipid-modifying agents (63 *versus* 70.2 per cent for intervention and standard care groups respectively; *P* = 0.297), or antiplatelets and/or anticoagulants (84 *versus* 85.7 per cent; *P* = 0.846) (*Table 3*).

### Self-reported adherence *versus* adherence according to the registry

**Fig. 4.**
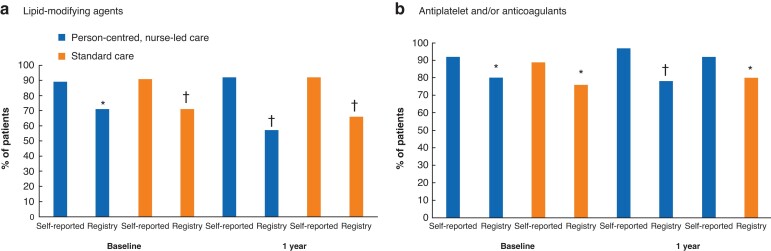
Proportion of patients with intake of medications for more than 6 days/week (proportion of days covered over 85 per cent) for lipid-modifying agents or antiplatelet and/or anticoagulant agents, according to self-reported data and data from prescribed drug registry from Centre for Health Data, Stockholm, Sweden **a** Lipid-modifying agents and **b** antiplatelet and/or anticoagulant agents. *P < 0.050, †*P* < 0.001 *versus* self-reported (related-samples McNemar test).

Self-reported adherence (PDC) to prescribed medication was higher than registry-based adherence in both groups, regardless of measurement time, allocation, or medication group (minimum *P* < 0.001, maximum *P* = 0.034). Self-reported high adherence (PDC over 85 per cent) was 92 per cent for lipid-modifying agents in the intervention group, whereas it was 57 per cent according to registry data (*P* < 0.001) (*Table S2* and *Fig. 4*).

### Secondary outcomes

#### Risk factors associated with cardiocerebrovascular disease

There was no difference in median FRS at 1 year between the intervention and control groups (both 18; *P* = 0.703). For the entire cohort, those who had quit smoking less than 6 months before surgery relapsed to a greater extent than those who had quit smoking more than 6 months before surgery (13 of 31 *versus* 7 of 144; *P* < 0.001). Regarding the treatment goal for HbA1c, there was no difference in improvement between the groups (*P* = 0.469). However, at 1 year, more patients in the control group were within the treatment goal for HbA1c level than in the intervention group (71 of 84 *versus* 57 of 81; *P* = 0.040). A similar tendency was seen at baseline (*Table 2*). No difference was noted between groups in low-density lipoprotein level (*P* = 0.880) or total cholesterol level (*P* = 0.168) at 1 year. For the total cholesterol level treatment goal, the intervention group deteriorated to a greater extent (12 of 75 *versus* 4 of 79; *P* = 0.034). A similar tendency was shown in deterioration from the treatment goal in low-density lipoprotein level (20 of 44 *versus* 13 of 47; *P* = 0.086).

As regards systolic BP, 39 of 93 patients in the intervention group reached the treatment goal compared with 32 of 98 in the control group. The corresponding numbers for diastolic BP measurement were 87 of 93 and 89 of 98 respectively. There were no differences between the groups (*Table 4*).

**Table 4:** Secondary outcomes: cardiocerebrovascular risk factors at 1 year after surgical treatment and change over time between baseline and 1 year

	Person-centred, nurse-led care (n = 99)	Standard care (n = 105)	P#
**Total Framingham risk score, median (i.q.r.) (*n*** **=** **88|90)**	18 (15 to21)	18 (16 to20)	0.703
* *Change (1 year – baseline), median (i.q.r.)	0 (−1 to 2)	0 (−1 to 2)	0.720
**Self-reported smoking status (*n*** **=** **98|102)**			0.878
Never smoker	8 (8)	8 (7.8)	–
* *Change (1 year – baseline)	0 (0)	0 (0)	–
Previous smoker	78 (80)	79 (77.5)	0.734
Current smoker	12 (12)	15 (14.7)	0.544
* *Relapsed to smoking (*n* =85|90)*†	8 (9)	12 (13)	0.481
**LDL (*n*** = **87|91)**			
Patients within treatment goal (≤ 1.8 mmol/l)	36 (41)	39 (43)	0.880
Deteriorated from treatment goal (*n* = 44|47)†	20 (46)	13 (28)	0.086
**Total cholesterol (*n*** = **89|93)**			
Patients within treatment goal (≤ 5 mmol/l)	70 (78)	81 (87)	0.168
Deteriorated from treatment goal (*n* =75|79)†	12 (16)	4 (5)	0.034
**HbA1c**			
Patients within treatment goal (≤ 52 mmol/mol with diabetes, ≤ 46 mmol/mol without diabetes) (*n* =81|84)	57 (70)	71 (85)	0.040
Reached treatment goal (*n* = 26|14)§	6 (23)	5 (36)	0.469
**Systolic BP (*n*** = **93|98)**			
Patients within treatment goal (< 140 mmHg)	39 (42)	32 (33)	0.231
Reached treatment goal (*n* = 55|69)§	19 (35)	16 (23)	0.228
**Diastolic BP (*n*** = **93|98)**			
Patients within treatment goal (< 90 mmHg)	87 (94)	89 (91)	0.594
Reached treatment goal (*n* = 9|7)§	8 (89)	3 (43)	0.106
**Pain-free walking distance**			
Patients with unlimited pain-free walking distance at 4–8 weeks after surgery (*n* = 97|88)	58 (60)	75 (85)	0.001
Patients with unlimited pain-free walking distance at 1 year after surgery (*n* = 93|95)	61 (66)	56 (59)	0.370
Deteriorated pain-free walking distance at 1 year after surgery (*n* = 55|71)¶	12 (22)	21 (30)	0.415
**ABPI (*n*** =**93|97), median (i.q.r.)**	0.90 (0.80 to1.00)	0.90 (0.70 to1.00)	0.701
Change (1 year – baseline), median (i.q.r.)	0.30 (0.14 to0.42)	0.27 (0.13 to0.43)	0.146
Change (1 year – 4–8 weeks after surgery), median (i.q.r.) (*n* = 91|97)	0.00 (−0.10 to 0.10)	0.00 (−0.11 to 0.10)	0.400
**BMI (kg/m^2^), mean(s.d.) (*n*** = **89|91)**	26.96(4.24)	27.38(4.90)	0.541
Change (1 year – baseline), median (i.q.r.)	−0.29 (−0.73 to 0.34)	0.00 (−0.35 to 0.91)	0.869
**Waist circumference (cm), mean(s.d) (*n*** = **91|90)**	101(10)	102(14)	0.441
Change (1 year – baseline), median (i.q.r.)	−2 (−6 to 1)	−1 (−6 to 2)	0.471

Values are *n* (%) unless otherwise indicated. Numbers of patients for whom data are available shown for intervention group|control group. *At 1 year, 13 of those who stopped smoking less than 6 months before inclusion relapsed compared with 7 who stopped smoking more than 6 months before inclusion.†Of patients within treatment goal at baseline. §Of patients out of treatment goal at baseline.¶Of those who had unlimited pain free-walking distance at 4–8 weeks after surgery. ABPI, ankle brachial pressure index; LDL, low-density lipoprotein. #Pearson χ2 or Fisher’s exact for proportions, the independent-samples Mann–Whitney U test, or the independent-samples test.

#### Ankle brachial pressure index

At 1 year, median ABPI was 0.90 (i.q.r. 0.80 to 1.00) and 0.90 (0.70 to 1.00) in the intervention and control groups respectively. There was a slight but non-significant improvement in the intervention *versus* control group compared with baseline) (*P* = 0.146) (*Table 4*).

#### Pain-free walking distance

Pain-free walking distance improved in both groups at 4–8 weeks after revascularization. A significantly higher proportion of patients in the control group had an unlimited walking distance than in the intervention group (75 of 88 *versus* 58 of 97 respectively; *P* = 0.001). However, this was no longer evident at 1 year (unlimited walking distance in 56 of 95 *versus* 61 of 93; *P* = 0.370). The control group seemed to deteriorate to a greater extent than the intervention group in walking distance at 4–8 weeks after revascularization (21 of 71 *versus* 1 of 55; *P* = 0.415) (*Table 4*).

The intervention group tended to have slightly lower mean BMI (*P* = 0.541) and waist circumference (*P* = 0.441) than with the control group at 1 year, but the differences were not significant.

## Discussion

Person-centred, nurse-led care, as performed in this study, had a similar effect and neither improved adherence to medication nor reduced risk factors for cardiocerebrovascular disease compared with standard care.

Adherence was expected to improve as a result of the intervention. Previous studies attempting an intervention to influence adherence have shown varying results. Granger *et al.*^13^, who identified non-adherent patients and intervened with a similar intervention reported a 20 per cent increase in the proportion of patients who took 80 per cent of pills (based on pill counts) compared with 5 per cent in the control group. In that study, the intervention was focused on influencing adherence to the medication regimen by providing information, setting up medication goals, facilitating medication–symptom associations, and using a symptom response plan^13^. An RCT^16^ that evaluated a rather focused intervention (pharmacist-led medication tailoring, patient education, collaboration between treating physicians, and educational and refill reminding voice messages) showed a significant improvement in adherence in all drug groups at 1-year follow-up after acute coronary syndrome. Conversely, another interventional study^36^, which evaluated the effect of telephone counselling in comparison with no telephone counselling among patients diagnosed with aortic aneurysm, peripheral arterial disease, or high BP, observed no effect of intervention in adherence to medication at 1- or 5-year follow-up. The intervention programme in the present study focused on information and involving the patient in planning to improve all aspects of secondary preventive measures and not only on adherence to the medication regimen, in contrast to that by Ho *et al.*^16^, and this study included all participants, not only those who were not adherent at baseline, in contrast to the study by Granger *et al.*^13^. It remains to be explored whether the subgroup of patients with low adherence to begin with would benefit more from the intervention in this study. As suggested by Conn and Ruppar^37^, pharmacist-delivered intervention and interventions focusing on habit-based (such as electronic reminders) behaviour-changing strategies, rather than on cognitive strategies aimed at changing knowledge and beliefs, may be an approach to maximizing adherence to medication.

One of the important results of the present RCT is that patients, when asked to self-report, overestimated their adherence to a medication regimen. Self-reported adherence to prescribed treatments differed from registry-reported adherence by between 12 and 35 percentage points. The method of asking patients whether they take medications has been questioned previously; in a meta-analysis^14^ of adherence to medication regimens after myocardial infarction, seven studies with self-reported data from a total of 49 791 patients showed a summary estimate of 90 (95 per cent c.i. 87 to 92) per cent adherence to a medication regimen. In the same meta-analysis^14^, studies using adherence measured by prescription refill frequencies reported a summary estimate of 57 (50 to 64) per cent adherence to a medication regimen. To the research group’s knowledge, no previous study has compared self-reported adherence with registry-reported data in the same study population. As patients’ recall of drug intake is an important source of information in clinical practice, it is vital to be aware of the probable overestimation of self-reported adherence to therapy. The results from the present study have shown that using self-reporting as a sole method to assess adherence is probably insufficient.

The mean adherence at 1 year of follow-up in the present study is greater than previously reported in a Swedish study^20^ with a similar population: approximately 75 per cent for lipid-lowering agents and 85 per cent for antiplatelet agents. Adherence was also greater compared with results from a meta-analysis^14^ that reported a summarized adherence of 66 per cent to antiplatelets and 76 per cent to lipid-lowering agents, based on studies that measured adherence using prescription refill rates. However, in the present study, prescribed doses were compared with filled prescriptions, resulting in calculation of actual patients’ adherence, whereas studies considering only filled prescriptions describe a combination of patients’ and physicians’ adherence (some of the patients may not be prescribed the drug), which may explain the perceived higher adherence results in this study. A large proportion of patients (over 90 per cent) had a prescription for both medication classes, antiplatelets and/or anticoagulants and lipid-modifying agents, compared with 65 per cent reported in other studies^20^. Guidelines^1^ published in 2017 emphasizing the benefits of lipid-modifying agents may explain the increase in the proportion of patients with a prescription for lipid-modifying agents.

The present findings, among others, have shown that adherence to secondary preventive medication must be improved. Kumbhani *et al*.^10^ reported that non-adherence to secondary prevention medications (antiplatelet, lipid-modifying, and hypertensive agents) is associated with an increased risk of all-cause mortality (number needed to treat 25). More research focusing on clinically applicable tools to identify non-adherence, to assess patients’ behaviour in taking prescribed secondary preventive medications, and to determine factors related to non-adherence is warranted.

Regarding secondary preventive measures other than medication, person-centred, nurse-led care showed no difference in reducing risk factors for cardiocerebrovascular disease compared with standard care. Similar results have been reported previously after extensive internet-based vascular risk factor management^38^. Kinmonth *et al.*^39^ showed no significant difference or worsening results after reviewing studies with patient-centred care interventions in patients with diabetes^38^. By contrast, another study^40^, with similar interventions to those described here offered to patients with acute coronary syndrome, reported a significant improvement in total cholesterol level, systolic BP, BMI, and physical activity. The discrepancy in results between the studies could be explained by the study populations because it is known that patients with cardiovascular disease are more likely to be adherent than those without^41,42^.

In the absence of positive results, the present study has, however, indicated that follow-up care of patients after surgical treatment for IC can be performed safely by nurses with experience in vascular surgery. Person-centred, nurse-led care may have an impact on sustainable lifestyle changes over time. In a previous qualitative publication from the FASTIC study^43^, the intervention group described more sustainable lifestyle changes. Data from the present study are consistent with this description as the intervention group tended to maintain smoking cessation to a greater extent. Considering the relatively high relapse rate among those who quit smoking less than 6 months before surgery, vascular surgery units may wish to reconsider the required duration of smoking cessation before revascularization for IC. Further studies addressing ways of increasing the sustainability of lifestyle changes are required.

Reports published during the study interval showed a surprisingly high adherence at baseline compared with the value used in the sample size calculation for the present study, which was based on earlier studies. With general adherence as high as in this study, an 20 per cent increase (as hypothesized) is not possible to achieve. However, the results are diverging, without any clear positive or negative trend in adherence for either the intervention or control group, leading to the deduction that a type II error is unlikely.

One of the main strengths of this study is the consideration of information on the number of days with prescriptions for studied medications, not only the number of days with available pills at home based on data on refill frequency. This makes the calculated patients’ adherence as accurate as possible without the confounding factor of physicians’ non-adherence. Nevertheless, because data on the number of days with a prescription were acquired from the start and end dates registered in the patients’ medical charts, inaccuracy of dates for the end of the prescription in the chart is a possibility for some patients. An example of this is where a physician informs the patient to stop a medication over the telephone or starts a new prescription with a similar medication without registering an end date in the medical chart. This may have contributed to some patients being classified as non-adherent; however, the issue applies to both the intervention and control groups. All measurements and clinical assessments were carried out in a routine clinical environment and were not standardized as in a research environment. This makes the data and the results interpretable and comparable with typical clinical data.

As there was no blinding during the planning or implementation of the study, the possibility of standard care being influenced by the intervention cannot be excluded. Physicians’ awareness of the study and its content may have led to different consultations about medications or risk factors than usual. Nevertheless, it is implausible for the complete contents of the intervention to be transferred to standard care, as the physicians did not attend courses on person-centred care, and were still limited to the duration and number of visits included in the standard care regimen.

The most common reason for choosing not to participate in the study was long distance to the hospital, even though the intervention involved only one extra visit to the outpatient clinic, which, if needed, could have been carried out by telephone. Offering other solutions, such as digital platforms, could be an alternative to maximize participation in studies of non-invasive interventions.

## Supplementary Material

znac241_Supplementary_Data
