# Supplementary material for: Effects of a person-centred, nurse-led follow-up programme on adherence to prescribed medication among patients surgically treated for intermittent claudication: randomized clinical trial
Source: Br J Surg. 2022 Jul 15;109(9):846–56. doi: 10.1093/bjs/znac241 (PMC10364713; doi:10.1093/bjs/znac241)
Supplement: znac241_Supplementary_Data [file znac241_supplementary_data.zip › Supplementary_Tables.docx]

Table S1: Type of vascular surgery performed, and complications.

|  | Person-centered,  nurse-led care  N =99 | Standard care  N =105 | P-value |
| --- | --- | --- | --- |
| Endovascular surgery/Hybrid  *(missing =36)* | 63/81 | 76/87 | 0.108 |
| Open Surgery/Hybrid  *(missing =36)* | 20/81 | 13/87 | 0.124 |
| Stents placed (above and below hip level)  (*missing =35)* | 48/82 | 60/88 | 0.206 |
| Dual antiplatelet therapy* | 26/99 | 34/105 | 0.540 |
| Surgical complications requiring treatment within 30 days | 5/99 | 1/105 | 0.208 |
| Surgical ipsilateral re-intervention within study year** | 12/99 | 14 /104 | 0.836 |

* Cardiac, post-stroke and post-surgical indications

** Three of all reoperated had CLTI (one from intervention group and 2 from control group)

Table S2: Proportion of days covered according to self-reported data and data from prescribed drug registry from Centre for Health Data, Stockholm, Sweden

|  | Baseline | | | | | 1 year | | | | | | | | |
| --- | --- | --- | --- | --- | --- | --- | --- | --- | --- | --- | --- | --- | --- | --- |
|  | Person-centered,  nurse-led care  N =99 | | | Standard care  N =105 | | | | Person-centered,  nurse-led care  N =99 | | | Standard care  N =105 | | | |
|  | Self-reported | Registry | P value | Self-reported | Registry | | P value | Self-reported | Registry | P value | Self-reported | Registry | P value |  |
| *Lipid-modifying agents* | *n =97* | *n =97* |  | *n =103* | *n =104* | |  | *n =88* | *n =97* |  | *n =88* | *n =104* |  |  |
| Intake of pills >6 days/w (PDC >85 %) | **86 (89)** | **70 (71)** | **0.003** | **94 (91)** | **74 (71)** | | **< 0.001** | **81 (92)** | **56 (57)** | **< 0.001** | **81 (92)** | **69 (66)** | **< 0.001** |  |
| Intake of pills ≥5 to 6 days/w (PDC ≥71% to 85%) | 1 (1) | 3 (3) |  | 1 (1) | 9 (9) | |  | 0 (0) | 13 (13) |  | 1 (1) | 9 (9) |  |  |
| Intake of pills <5 days/w (PDC <71%) | 1 (1) | 24 (24) |  | 2 (2) | 21 (20) | |  | 2 (2) | 28 (28) |  | 2 (2) | 26 (25) |  |  |
| Have no prescription or don’t know if they have | 9 (9) | 2 (2) |  | 6 (6) | 1 (1) | |  | 5 (6) | 2 (2) |  | 4 (5) | 1 (1) |  |  |
| *Antiplatelets and/or anticoagulants* | *n =97* | *n =99* |  | *n =104* | *n =105* | |  | *n =88* | *n =99* |  | *n =89* | *n =105* |  |  |
| Intake of pills >6 days/w (PDC > 85 %) | **89 (92)** | **79 (80)** | **0.031** | **92 (89)** | **80 (76)** | | **0.031** | **85 (97)** | **77 (78)** | **< 0.001** | **82 (92)** | **84 (80)** | **0.034** |  |
| Intake of pills ≥5 to 6 days/w (PDC ≥71% to 85%) | 0 (0) | 10 (10) |  | 1 (1) | 7 (7) | |  | 0 (0) | 10 (10) |  | 0 (0) | 12 (12) |  |  |
| Intake of pills <5 days/w (PDC <71%) | 0 (0) | 10 (10) |  | 4 (4) | 18 (17) | |  | 0 (0) | 12 (12) |  | 3 (3) | 9 (9) |  |  |
| Have no prescription or don’t know if they have | 8 (8) | 0 (0) |  | 7 (7) | 0 (0) | |  | 3 (3) | 0 (0) |  | 4 (5) | 0 (0) |  |  |

PDC =Proportion of days covered. Values are presented in numbers (percentages).
